# Optimal Waveforms Design for Ultra-Wideband Impulse Radio Sensors

**DOI:** 10.3390/s101211038

**Published:** 2010-12-06

**Authors:** Bin Li, Zheng Zhou, Weixia Zou, Dejian Li, Chong Zhao

**Affiliations:** 1 Key Lab of Universal Wireless Communications, Ministry of Education (MOE), Inner Box. 96, BUPT, Beijing 100876, China; 2 Beijing University of Posts and Telecommunications (BUPT), Inner Box.96, BUPT, Beijing 100876, China; E-Mails: zzhou@bupt.edu.cn (Z.Z.); zwx0218@bupt.edu.cn (W.X.Z.); dejian021@163.com (D.J.L.); Emmacinderey@gmail.com (C.Z.)

**Keywords:** UWB-IR sensors, waveform design, orthogonality, waveform division multiple access

## Abstract

Ultra-wideband impulse radio (UWB-IR) sensors should comply entirely with the regulatory spectral limits for elegant coexistence. Under this premise, it is desirable for UWB pulses to improve frequency utilization to guarantee the transmission reliability. Meanwhile, orthogonal waveform division multiple-access (WDMA) is significant to mitigate mutual interferences in UWB sensor networks. Motivated by the considerations, we suggest in this paper a low complexity pulse forming technique, and its efficient implementation on DSP is investigated. The UWB pulse is derived preliminarily with the objective of minimizing the mean square error (MSE) between designed power spectrum density (PSD) and the emission mask. Subsequently, this pulse is iteratively modified until its PSD completely conforms to spectral constraints. The orthogonal restriction is then analyzed and different algorithms have been presented. Simulation demonstrates that our technique can produce UWB waveforms with frequency utilization far surpassing the other existing signals under arbitrary spectral mask conditions. Compared to other orthogonality design schemes, the designed pulses can maintain mutual orthogonality without any penalty on frequency utilization, and hence, are much superior in a WDMA network, especially with synchronization deviations.

## 1. Introduction

Ultra-wideband impulse radio (UWB-IR) is a promising technique in short-range high-data-rate communication scenarios, such as wireless personal area networks (WPANs) [1]. Meanwhile, UWB-IR sensors have also been employed in military applications such as high-precision radar and through-wall target detection owing to their exceptional multipath resolution and material penetration capability [2–5]. Most recently, the emerging body area network (BAN) field also considers UWB as an appealing solution for health monitoring. These advantages of UWB-IR are mainly attributed to the enormous bandwidth of its transmitted pulses, which may occupy several gigahertz (GHz). However, on the other side, UWB also has long been confronted with rigorous application restrictions, because of its potential interference to other existing vulnerable wireless systems, such as Global Positioning System (GPS) and Universal Mobile Telecommunications System (UMTS) [6]. The first UWB emission mask was set out by U.S. Federal Communications Commission (FCC) in 2002, accompanying the authorization of its unlicensed use in the 3.1–10.6 GHz band [7].

For thorough spectral compatibility between those systems sharing the same band, the released UWB emission limits are very strict; for example, the FCC allowable equivalent isotropically radiated power (EIRP) for UWB transmitted signals is below −41.3 dBm/MHz. Hence, with respect to this EIRP mask, only when the transmitted pulses make full use of the regulated spectral energy, can a sufficiently high signal to noise ratio (SNR) be obtained in UWB receivers, which in turn enhances transmission reliability. Although the traditional Gaussian monocycle has been widely used in the early stages because of its simple realization, its frequency utilization is quite limited [8], so many publications have focused on this issue in recent years. In [9,10], Parr constructed an equivalent channel matrix from the sampled mask, and generated orthogonal UWB pulses from its dominant eigenvectors. However, the frequency utilization remains rather low, and the required 64 GHz sampling frequency makes it comparatively hard to implement. The Finite Impulse Response (FIR) filter based technique adopting Parks-McClellan (PM) algorithm has been presented in [11]. Unfortunately, the spectral mismatch between the designed PSD and emission mask is remarkable near the sharp spectral discontinuities. Davidson *et al.* [12] applied linear matrix inequalities (LMI) theory to design FIR filter, which could conform to piecewise constant and piecewise trigonometric polynomial masks. Later, a FIR-based pulse shaper has been fully extended by using second order cone programming (SOCP) and it achieved relatively high frequency utilization [13,14]. However, their expected filter orders may be comparative large in order to achieve an acceptable frequency utilization, and the pulses still cannot use the lower frequency region (0–0.9 GHz) entirely. In [15], Ohno and Ikegami synthesized an interference mitigation waveform. Such a UWB pulse can use one single band only and its realization is very complicated given the dozens of carrier generators required both in transmitters and receivers. Other UWB waveform optimization techniques, such as the optimal waveform designing based on Gaussian functions or Rayleigh functions, can match the whole spectral mask to some extent [16,18]. Nevertheless, the frequency utilization of these optimal pulses is still far from satisfactory.

In addition, modern communication design has gradually paid attention to resolving the spectrum scarcity, so that the orthogonal waveform multiplexing have been widely adopted to further improve the frequency efficiency, which can also eliminate mutual interference or provide considerable waveforms diversity gain in UWB sensor networks [19]. Therefore, an orthogonal waveform set becomes indispensable in system design. The Hermite-Gaussian function and wavelet have been introduced to design mutually orthogonal UWB waveforms; however, their frequency utilization cannot been further optimized [20,21]. Although spectrally efficient orthogonal waveforms have been devised based on the FIR filter [13,14], the complexity of this sequential algorithm may grow with the increasing number of orthogonal users. More importantly, the frequency utilization of subsequent derived pulses undergoes an obvious degradation. Besides, these designed orthogonal pulses are rather sensitive to synchronization deviations, which imposes stringent requirements on receiving timing and hence increases complexity [14,20].

In this paper, we propose a novel pulse forming technique for UWB-IR sensors. The frequency domain representation of the emission pulse is firstly derived from the product of a weight vector and the cyclic shift matrix (CSM) constructed from the basis waveforms. As a result, the spectral shaping problem is transformed to an optimization of the corresponding weight vector. With the permission that the designed PSD can temporarily outstrip UWB spectral masks, the design process can be simplified greatly. Later, this preliminary waveform would be further modified iteratively to lower the excess PSD until UWB pulses totally conform to emission constraints. Numerical evaluations indicate that our pulse can match the arbitrary spectral constraint much more completely than the other existing schemes. The proposed structure can also be viewed as a versatile pulse generator which can be efficiently implemented for digital signal processing (DSP). Hence, it can be directly applied to arbitrary UWB masks. We also design UWB waveforms with spectrum notch attenuated nearly 50 dB in specific bands, which is of great significance for cognitive radios (CRs) considering spectral avoidance to primary users.

Based on this already proposed algorithm, the constraint on orthogonal waveforms has also been derived. In order to obtain orthogonal pulses, schemes both from time domain and frequency domain have been addressed. We demonstrate that our designed orthogonal waveforms can use spectral mask as entirely as a single pulse. It is shown through analysis and simulation evaluations that the designed orthogonal pulses outperform other UWB waveforms in a WDMA network if mutual interference from nearby sensors is taken into account, especially when the synchronization deviation exists.

The rest of this paper is organized as follows: Section 2 elaborates on the design algorithm in detail. The orthogonal UWB pulses with efficient frequency utilization will be analyzed in Section 3. In Section 4, we discuss and evaluate the performance of UWB pulses in WDMA network with different degree of timing accuracy. At last, we conclude the paper in Section 5.

## 2. UWB Waveform Design

In order to eliminate potential interference from UWB sensors to the other vulnerable wireless systems sharing the same frequency band, the emission power of transmitted UWB pulses has been rigorously limited in different frequencies [6]. The regulatory FCC spectral mask for indoor UWB devices can be shown as:

(1) $$M_{\text{FCC}}\left(f\right)=\{\begin{array}{ll} -41.3\;\text{dBm}/\text{MHz} & f\in \left[0,\;\;0.96\right]\text{GHz}\\ -75.3\;\text{dBm}/\text{MHz} & f\in \left[0.96,\;\;1.61\right]\text{GHz}\\ -53.3\;\text{dBm}/\text{MHz} & f\in \left[1.61,\;\;1.99\right]\text{GHz}\\ -51.3\;\text{dBm}/\text{MHz} & f\in \left[1.99,\;\;3.1\right]\text{GHz}\\ -41.3\;\text{dBm}/\text{MHz} & f\in \left[3.1,\;\;10.6\right]\text{GHz}\\ -51.3\;\text{dBm}/\text{MHz} & f\in \left[10.6,\;\;+\infty \right]\text{GHz} \end{array}$$

Thus, UWB sensors in preparation for data transmissions should make sure that their power spectrum density (PSD) remains below *M_FCC_*(*f*). For the time-hopping pulse position modulation (TH-PPM) and pulse amplitude modulation (TH-PAM) based multiple-access system, the accumulated PSD of multiusers can be approximated well by *K|S*(*f*)*|*^2^ [11,13,22]. *S*(*f*) represents the Fourier transform (FT) of baseband pulse *s*(*t*), while *K* is a constant related to the specific time-hopping (TH) code [22], which has no relation with the pulse designing. To simplify elaborations, we directly set *K* = 1 in our following analysis so the designed pulse should satisfy an important confinement *|S*(*f*)*|*^2^ ≤ *M_FCC_*(*f*). When we adopt a more *general* emission limits, denoted by *M*(*f*) which is regulated by different countries, the corresponding confinement can be further modified to *|S*(*f*)*|*^2^ ≤ *M*(*f*).

To improve SNR in receivers, on the other hand, the transmitted UWB pulse is also supposed to use the regulatory spectral power as fully as possible. The spectral utilization efficiency of UWB signals is always measured in terms of the normalized effective signal power (NESP) [13], which is defined as:

(2) $$\text{NESP}=\int_{f_{B}}|S\left(f\right){|}^{2}\text{df}/\int_{f_{B}}|M\left(f\right)|\text{df}\times 100\%$$

Where *M*(*f*) is the spectral mask regulated by the radio management and *f_B_* denotes the authorized band. As a consequence, the consolidated objective of UWB waveform designing is to maximize the NESP subject to *|S*(*f*)*|^2^* ≤ *M*(*f*). Traditionally, the NESP optimization is mainly focused on two techniques, that is, the UWB pulse shaping filter design and waveform optimization. Basically, both two techniques can only concentrate on devising appropriate time sequences which are expected to exhibit specific spectrum shapes, including the impulse response of FIR filters and UWB waveforms. These design methods are usually either complicated in their realizations or inefficient in NESP. In this paper, our new scheme will handle the UWB signal design directly in the transform domain, which is much more competitive from the aspect of its substantivity of maximizing the NESP.

### 2.1. Design Algorithm

To begin this goal-directed design algorithm, we may select specific waveform meeting the following two restrictions as the basis waveform in frequency domain:

1. The basis waveform should be symmetric. Actually, the symmetry waveform is much suitable in the sense that the UWB spectrum mask remains constant in most frequency range. Besides, it is easy to generate an even symmetry waveform from the classical FIR filter [23].
2. The basis waveform is also supposed to attenuate fast. This is mainly because the regulatory UWB spectral masks always contain *sharp stairs* or *narrow notches*, whereas the long trailing of basis waveform may destroy the chop features of UWB mask.

**Figure 1.**
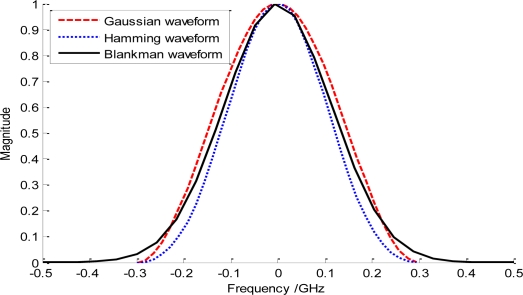
Basis waveforms meeting the requirements.

In general, the energy concentration of the basis/windowing waveforms can be used to essentially reflect their attenuation characteristic, which can be usually defined as *∫_f_*_1_*|w*(*f*)*|*^2^*df*/*∫_fw_|w*(*f*)*|*^2^*df*. Here, *w*(*f*) is the basis waveform in frequency domain; *f*1 represents frequency range limited by the −10 dB cutoff points, while *fw* denotes the interested frequency ranges. It is clearly seen that the higher the energy concentration is, the faster the waveform attenuation is. So, the conventional Gaussian waveform and the raised cosine function are both good candidates for the basis waveforms, whose energy concentrations can basically approach 99.8% and 99.4%, respectively. Some familiar basis waveforms meeting the challenges have been shown in Figure 1.

However, it should be noted that although the rectangular waveform has an ideal energy concentration, the corresponding infinite waveform in time domain may prevents it from being applied. In our following analysis, we adopt the Gaussian monocycle as the basis waveform because of its simple realization on hardware. So, we have:

(3) $$w\left(f\right)=\frac{1}{\sqrt{2\pi s}}\text{exp}\left(-\frac{f^{2}}{2s^{2}}\right)$$

where *s* is the shaping parameter which determines the waveform shape, including the height and width of *w*(*f*). The corresponding sample basis sequence *w*(*k*) can be given by:

(4) $$w\left(k\right)=\{\begin{array}{cc} w\left(f\right){|}_{kf_{s}} & k=0,1,\ldots ,N/2-1\\ w\left(f-{\text{Nf}}_{s}\right){|}_{kf_{s}} & k=N/2,N/2+1,\ldots ,N-1 \end{array}$$

where *f_s_* represents the sample interval in frequency domain; *N* is the length of the basis sequence. A cyclic shift matrix (CSM), **W**, can be then constructed from this basic sequence *w*(*k*) above:

(5) $$\text{W}=\left[\begin{array}{cccc} w\left(0\right) & w\left(N-1\right) & \cdots & w\left(1\right)\\ w\left(1\right) & w\left(0\right) & \cdots & w\left(2\right)\\ \vdots & \vdots & \ddots & \vdots \\ w\left(N-2\right) & w\left(N-3\right) & & w\left(N-1\right)\\ w\left(N-1\right) & w\left(N-2\right) & & w\left(0\right) \end{array}\right]$$

We denote the first column of **W** with **w**_0_:

(6) $${\text{w}}_{0}={\left[w\left(0\right)\;w\left(1\right)\cdots w\left(N-2\right)\;w\left(N-1\right)\right]}^{T}$$

After the cyclic shift of *i* samples have been performed on **w**_0_, we have:

(7) $${\tilde{\text{w}}}_{i}={\tilde{\text{w}}}_{0}\left(k-i\right)={\text{w}}_{0}{\left(\left(k-i\right)\right)}_{\text{N}},\;k=0,1,\cdots N-1$$

Here, the notation **w**_0_((*k*-*i*))*_N_* represents the *i* samples cyclic shift operation on **w**_0_ [23]. Accordingly, **W** can be expressed into a much compact form:

(8) $${\text{W}}_{N\times N}=\left[{\tilde{\text{w}}}_{0}\;{\tilde{\text{w}}}_{1}\cdots {\tilde{\text{w}}}_{N-1}\right]$$

From (8), it is clear that each column of the cyclic shifted matrix **W** represents a basis waveform with its center located at different frequency band. If an appropriate weight vector **a** has been selected, according to the *interpolation theory*, the weighted sum of these elements would match the spectral mask at different bands. This weight vector **a** is a column vector with *N* elements:

(9) $$\text{a}={\left[\alpha_{0}\;\alpha_{1}\cdots \alpha_{N-1}\right]}^{T}$$

As a result, we obtain the frequency domain representation of UWB pulse conveniently, from the product of the optimized weight vector **a** and the shifted matrix **W**:

(10) $$S\left(k\right)={\text{a}}^{T}\text{W}=\sum_{i=0}^{N-1}\alpha_{i}{\tilde{\text{w}}}_{i}$$

In order to maximize NESP, from (10), optimization should be performed on the weight vector **a**. Actually, maximizing the NESP will be equivalent to minimizing the mean square error (MSE) between the obtained power spectrum density *|S*(*f*)*|^2^* and the FCC mask *M_FCC_*(*f*), if we postulate that the designed PSD would conform to the whole emission mask. In fact, however, the rationality of above equivalency can not be always guaranteed and the designed PSD will exceed the emission limit during certain narrow ranges. All the same, our design algorithm can be composed of *two phases*. The inevasible excess PSD is permitted in the first phase in order to simplify solving process, and therefore, the UWB pulse is obtained by only minimizing the MSE. Then, in the second phase, the already generated waveform would be further modified to eliminate the residual interference in mismatched band. In particular, the optimal weighting vector **a** can be firstly obtained from:

(11) $${\text{a}}_{\text{opt}}={\text{arg}\;\text{min}\left\|S^{2}\left(f\right)-M\left(f\right)\right\||}_{f=kf_{s}}=\text{arg}\;\text{min}\left\|{\left(\sum_{i=0}^{N-1}\alpha_{i}w_{i}\right)}^{2}-M\left(k\right)\right\|$$

It is obvious that the objective function in (11) is a *concave* surface for the weight vector **a**. So the optimal solution **a***_opt_,* which minimizes the MSE between *|S*(*f*)*|^2^* and *M_FCC_*(*f*), is supposed to exist uniquely. Therefore, those classical iterative algorithms, such as the steepest descent algorithm (e.g., LMS algorithm) [24, 25], can be effectively employed to achieve the convergence of the optimal weight vector **a***_opt_*:

(12) $${\text{a}}_{\text{opt}}={\text{M}}_{k}{\text{W}}^{-1}$$

where **M***_k_* is a vector composed of the sampled spectral mask. If the algorithm complexity is taken into consideration, the solution of (12) may be computationally expensive because of the large dimension of **W**. Nevertheless, the dimension of **W** can be reduced if the cyclic shift factor is chosen to be larger than 1. Thus, the solving process and the hardware implementation of the proposed scheme are greatly simplified. The dimension reduced cyclic shift matrix **W**_↓_ can be rewritten as:

$${\text{W}}_{↓}=\left[\begin{array}{cccc} w\left(0\right) & w\left(N-1\right) & \cdots & w\left(N-ml\right)\\ w\left(1\right) & w\left(N-l+1\right) & \cdots & w\left(N-ml+1\right)\\ \vdots & \ddots & & \vdots \\ w\left(N-2\right) & w\left(N-l-2\right) & \cdots & w\left(N-ml-2\right)\\ w\left(N-1\right) & w\left(N-l-1\right) & \cdots & w\left(N-ml-1\right) \end{array}\right]$$

where *l* (*l* > 1) represents the cyclic shift factor; *m* is equal to ⌊*N/l*⌋ which can be viewed as the *orders of equivalent filter*. In fact, Equation (12) is based on the assumption that matrix **W** is a *square matrix*. So the inverse matrix can be directly employed during the derivation of weighting vector. However, notice that the dimension-reduced matrix **W**_↓_ is now a non-square matrix. Accordingly, the inverse matrix in (12) can be replaced by the *pseudo-inverse* of the *N* × *l* dimensional matrix **W**_↓_ [23]. Then, the optimal weight vector **a***_opt_* is modified to:

(13) $${\text{a}}_{\text{opt}}={\text{M}}_{\text{k}}{\left({\text{W}}_{↓}^{\text{T}}{\text{W}}_{↓}\right)}^{-1}{\text{W}}_{↓}^{\text{T}}$$

At the expense of the complexity reduction, slight fluctuations will appear in the flat part of spectral mask and the smooth transitions will replace the sharp discontinuous edges in the designed PSD, which may reduce the obtained NESP. In most cases, however, this compromise between the complexity and the NESP is worthwhile, especially when the downside influence on NESP is insignificant.

Although the output pulse based on (12) can meet the spectral constraint in most frequency bands, it is also noteworthy that the designed PSD has exceeded the emission limit during the narrow range near the sharp discontinuous edges. From the numerical simulation shown in Figure 3(a), the maximum excess can even reach 20 dB under the FCC mask. For UWB emission limits with the abrupt slope shape, such as the ECC spectral limit [26], this mismatch near 0–2.5GHz is considerably serious in unmodified UWB pulse. The previous ECC regulated mask *M_ECC_*(*f*) for outdoor applications can be given by [26]:

(14) $$M_{\text{ECC}}\left(f\right)=\{\begin{array}{cc} -61.3+87\text{log}\left(f/3.1\right)dB & f<3.1Hz\\ -41.3dB & f\in \left[3.1,\;\;10.6\right]GHz\\ -61.3+87\text{log}\left(10.6/f\right)dB & f>10.6GHz \end{array}$$

It is noteworthy that the slope mask is in *logarithmic scale* instead of linear scale, as is indicated by [26]. Definitely, this overlarge excess PSD obtained by only minimizing MSE will introduce serious interference to other vulnerable wireless services occupying the corresponding band [27]. Thus, it is absolutely necessary to further modify the original output UWB pulses before they can be practically applied under the safety constraint *|S*(*f*)*|*^2^ ≤ *M_FCC_*(*f*). Inspired by this conceptual method, we can adjust a small part of the already optimized weight vector **a***_opt_* in order to further control the serious interference from excess PSD. We denote the subset of weight vector by **a**_mod_ which corresponds to the mismatched band [*f_dowm_* *f_up_*]:

(15) $${\text{a}}_{\text{mod}}=\left[a_{i}\;a_{i+1}\cdots a_{j}\right]$$

The subscript in (15) is determined by:

(16) $$i=\left\lfloor f_{\text{down}}/\left(l\times f_{s}\right)\right\rfloor ,\;j=\left\lceil f_{\text{up}}/\left(l\times f_{s}\right)\right\rceil$$

So, the subset **a***_mod_* associated with these mismatched ranges can be easily determined from (16) after minimizing the MSE, and then, it will be further updated iteratively according to:

(17) $$a_{i}^{\left(k\right)}=\{\begin{array}{ll} \lambda a_{i}^{\left(k-1\right)} & i\in \cup_{n=1,2,3,4}{f\_\text{set}}_{n}/{\left(l\times f_{s}\right),\;S^{2}\left(f\right)-M\left(f\right)|}_{f={f\_\text{set}}_{n}}\geq ɛ\\ a_{i}^{\left(k-1\right)} & i\in \cup_{n=1,2,3,4}{f\_\text{set}}_{n}/{\left(l\times f_{s}\right),\;S^{2}\left(f\right)-M\left(f\right)|}_{f={f\_\text{set}}_{n}}\leq ɛ\\ a_{i}^{\left(k-1\right)} & i\notin \cup_{n=1,2,3,4}{f\_\text{set}}_{n}/\left(l\times f_{s}\right) \end{array}$$

where *f*_*set_n_* represents the *n^th^* frequency range where the designed PSD surpasses the emission mask. *λ* is an *updating step* which is always around 0.9–0.99; *ɛ* is the *tolerable interference* which is chosen to be 0.1 dB in our analysis for the purpose of fast convergence. For some realistic applications, however, *ɛ* should be strictly set to 0 dB to completely mitigate interference. This iteration modification can be initialized by the already converged optimal vector **a***_opt_* obtained from (11). This process is continued until *S*^2^(*f_set_n_*)*-M_FCC_*(*f_set_n_*)≤*ɛ* has been completely fulfilled. Given the mismatch under ECC mask mainly appears in extremely abrupt slope where the emission limit always remains below—90 dBm/MHz, for example in ECC spectral mask, we may alternatively reset **a***_mod_* to eliminate the serious interference, which means for each *α*(*i*) that fall in the spectrally excessive ranges determined from (16), we may directly let *α*(*i*) = 0.

Now, we investigate the convergence property of this iterative updating process. In fact, much similar to the Gibbs phenomenon encountered in most *Fourier series approximation* problems under the minimum MSE criterion, the maximum excessive spectrum value near the sharp edges can be approached in practice by a constant*,* which can be denoted by Δ. On the other hand, if the number of basis functions is large enough, it is obvious that the weight components, *α*(*i*), may have an asymptotic convergence to $\sqrt{M(i)}$. Therefore, when we decrease the weight component step by step, then the corresponding spectral value will also be reduced gradually. If we denote the updating step by *λ*, then the UWB waveform totally complying with the spectral limitation can be produced after around ⌈Δ/*λ*⌉ iterations which is also a constant, regardless of the specific emission mask and the basis functions. So, the fast convergence of the iterative algorithm can be basically guaranteed, with a linear complexity of *O*(*m*) given the spectral constraint *M*(*k*), where *m* is the total number of weight components that is near the spectral discontinue edges. Furthermore, for most realistic spectral mask containing few sharp edges/discontinuity (usually smaller than seven spectral edges), this computational expense can be basically ignored.

It is also noted that, from (11), the optimization formulation is essentially a convex problem. Hence, the adopted LMS algorithm can definitely find the minimum MSE solution. Alternatively, the first stage can be also directly realized by resorting to numerical computation based on (13). Consequently, given that the convergence of both the two phases can be guaranteed, this whole proposed algorithm can also converge to the optimal solution after the finite iterations.

### 2.2. UWB Pulse Generation

To sum up the points which we have just indicated, our proposed designing algorithm contains the following two phases:

*Phase 1*: Based on (11), the optimized weight vector **a***_opt_* can be easily obtained. Focusing on the minimizing of the *MSE* between *S*^2^(*f*) and *M*(*f*), unfortunately, the generated pulse would inevitably excess the UWB emission mask in certain frequency range, which is mainly caused by the unreasonable equivalence of (11).

*Phase 2*: The excess PSD may lead to awful interference to other wireless services. Hence, the main purpose of this phase is to prune the excess PSD at the expense of slightly decreasing the already maximized NESP. The subset of weight vector exactly corresponding to the undesirable interference will be further modified. This modification process can be basically concluded into two rules: (1) For the output pulse with the excess PSD located at the extremely narrow band, or the band has a serious attenuation from the maximum allowable EIRP, we may directly reset the corresponding subset **a***_mod_*. (2) For the others, we adopt the iterative process in (17).

After the spectrum pruning process, the UWB pulse can be immediately derived by the inverse discreet Fourier transform (IDFT) on *S*(*k*):

(18) s(n)=IDFT(S(k)⊗exp(jθ))

where **a**⊗**b** represents the product of two vectors, whose component is the product of the corresponding two elements of **a** and **b**. The column vector **θ** represents the user defined phase response of UWB pulse which is usually chosen to be a linear phase. By substituting (10) into (18) and applying the *cyclic shift property* of DFT, a much explicit form of *s*(*n*) can be shown as:

(19) $$s\left(n\right)=\sum_{i=0}^{N-1}\alpha_{i}w^{\prime }\left(n\right)\text{exp}\left(-j\frac{2\pi }{N}\text{nil}\right)$$

where *w*’(*n*) is IDFT of the basis sequence *w*(*k*). The generated UWB waveform from (19) is complex. As is well known, the Fourier transform of a conjugate symmetric sequence is real. So we can construct the even symmetric component *S_even_*(*k*) from *S*(*k*) to further design the real waveform.

(20) $$S_{\text{even}}\left(k\right)=\frac{1}{2}S\left(\left|k\right|\right)\;\;\;k=-N+1,\cdots ,0,1,\cdots N-1$$

Also, the phase response should be specified to be a linearly odd function of *k*. Then, if *the even and odd signals and spectra property* of the DFT is applied [23,27], the real UWB pulse can be derived. Accordingly, the Fourier transform of this real waveform includes two parts that remain *conjugate symmetry* [23], and the amplitude response of each part is in direct proportion to *S*(*k*).

(21) $$s_{\text{real}}\left(n\right)=\text{IDFT}\left(S_{\text{even}}\left(k\right)\right)=\sum_{i=0}^{N-1}\alpha_{i}w^{\prime }\left(n\right)\text{cos}\left(-i\frac{2\pi }{N}\text{ln}\right)$$

### 2.3. Implementation

**Figure 2.**
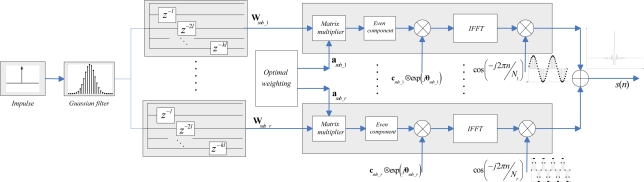
Structure of proposed UWB pulse generator. Note that the *Even component* modular constructs *S_even_*(*k*) from *S*(*k*). The term **c***_sub__*_1_⊗*exp*(j**θ***_sub_1_*) denotes the vector product of user defined phase response and orthogonal codes. For single UWB waveform design, **c** = **1**^1×^*^N^*. Also, notice that when *r* = 1, the carrier shifting process can be *avoided*.

The descriptive structure of the proposed UWB pulse is illustrated in Figure 2. First, an impulse sequence with period of *N* samples is generated. This impulse signal is fed into a Gaussian shaping filter with *BT* = 0.4 and then the basis sequence *w*(*k*) is formed. To simplify the hardware realization, especially to reduce the baseband sampling frequency, we divide the total UWB band evenly into *r* subbands and further employ *r branches* circuit to generate each subband signal corresponding to the frequency band [*f_i_down_* *f_i_up_*], *i=*0,1,…,*r-*1. In the *i^th^* branch, the cyclic shifted matrix **W**_s_*_ub_i_* can be constructed after the sample sequence *w*(*k*) passed a *cyclic shift modular*. Considering the periodicity of the input impulse sequence, as is illustrated in Figure 2, these cyclic shift modules can be realized by *a group of delays* with a delay factor *kl,* where *k* is an integer whose value ranges from 0 to ⌊*N/lr*⌋.

The expression of subband UWB signal *S_sub_i_*(*k*) in frequency domain would emerge from the matrix product between **W**_s_*_ub_i_* and the optimal weight vector **a***_sub_i_* based on (10). Then, the corresponding UWB waveform can be produced by *IDFT* on the symmetric component of *S_sub_i_*(*k*). Multiplying each brand signal by a single carrier with a center frequency of *f_i_* and summing them up, the UWB pulse occupied the whole band can be finally formed:

(22) $$f_{i}=\left(2i-1\right)f_{\text{max}}/2r$$

where *f_max_* denotes the designed maximum frequency of UWB signals. The desired sampling frequency in the baseband process is determined by the maximum sampling rate of each branch. If the designed maximum frequency is 12.5 GHz and we divide the total band equally into two subbands, the *baseband sampling rate* is only about 7 GHz according the sampling theory of baseband signals [23], which is much lower than the desired sampling frequency of 28 GHz in the SOCP method [13,14]. Given the primary *hardware barrier* lies in the very precise delay lines at high baseband sampling rate, our scheme is much more facilitative of implementation. It is worth noting that the main processes of this structure are the samples *delay* and IDFT. Considering that the fast Fourier transform (FFT) algorithm has been widely embedded in modern communication systems and the adaptive algorithm (e.g., LMS algorithm) of the weight vector **a** also has an efficient realization, our proposed pulse is rather simple and effective compared with the pulse generator in [14], which requires lots of carrier generators to synthesize UWB waveform. In addition, this structure can be used as a versatile *UWB shaper filter*, which means it can be directly expanded to another emission mask without any modification either on hardware or algorithm. It is emphasized that when *r* = 1, *the carrier shifting process can be avoided accompany the carrier generator*, at the expense of high baseband sampling rate as in [13] and [14].

It is observed that, from Figure 2, if the single carrier with a center frequency of *f_i_* can be accurately synthesized, the cyclic shift matrix **W** (or **W**_↓_) is *unique* to different values of *r*, according to the frequency shifting property of IDFT [23,33]. Therefore, when the whole branches number changes, the generated UWB waveforms may still remain similar to each other, given the spectral mask and the basis functions number *n*. On the other hand, this alterative implementation structure is also quite immune to carrier synthesis errors. In practice, a slight shifting in the carrier signal may have a serious effect on the time domain waveform (e.g., the time duration) as well as the frequency domain spectrum (e.g., the spectral mismatch caused by the over staggered basis functions). This situation may be further deteriorated with the increasing of *r*. As a result, a large branches setting may greatly alleviate the impractical requirement on high-speed ADC devices, but also pose great challenge in carrier synthesizers. For most practical applications, the hardware architecture with two subbands seems to be a reasonable compromise considering the effect on both baseband sampling rate and required carrier accuracy.

### 2.4. Spectrum Utilization

**Figure 3.**
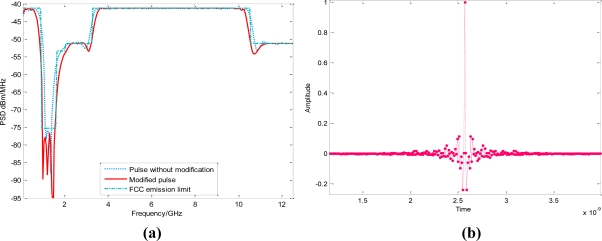

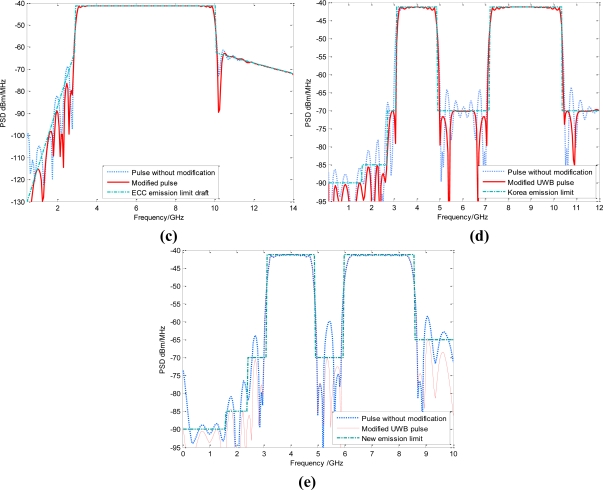
**(a)** Designed UWB waveform under FCC emission mask. The dimension of **W**_↓_ is 32 × 128. Notice that the modified UWB pulses can now totally comply with emission limits. **(b)** Time domain waveform under FCC mask. **(c)** Designed UWB pulse under ECC emission mask. The dimension of **W**_↓_ is 48 × 128. **(d)** Designed UWB pulse under Korea emission mask. The dimension of **W**_↓_ is 48 × 128. **(e)** Designed pulse under the new ECC emission mask. The dimension of **W**_↓_ is also 48 × 128.

In our simulation, the maximum working frequency of UWB pulse is set to 12.5 GHz; the basis waveform length *N* is 128. The cyclic shift factor *l* is 8, so the equivalent filter orders are 32 which actually correspond to the length of the weighting vector. The UWB pulse under FCC mask can be designed as is illustrated in Figure 3(a). From the simulation results, UWB waveforms designed just from the first phase, without the further modification process, may surpass FCC emission mask near the following four narrow frequency bands: *f_set*_1_ = [0.96,1.12], *f_set*_2_ = [1.43,1.6], *f_set*_3_ = [2.95,3.1], *f_set*_4_ = [10.6–10.76]. However, the modified PSD |*S*(*f*)|*^2^* can completely comply with the FCC spectral mask. Similar spectral mismatch can be observed from the other masks.

Meanwhile, it is noted from Figure 3(c) that the NESP of the modified UWB waveforms under ECC mask is quite close to that of the original output PSD from (11). This is because the removed spectra energy is usually insignificant.

Clearly, this UWB pulse can also entirely utilize the frequency band below 1GHz. Although slight mismatches appear near the spectral sharp discontinuous edges in original MSE-based algorithm, the obtained NESP of modified pulses is quite encouraging. For UWB waveform that totally keeps below FCC spectral mask, the NESP can even reach 98.71% when the dimension of **W**_↓_ is 32 × 128.

Although the FCC spectral limit *M_FCC_*(*f*) has been used as the design objective in our above elaborations, as is mentioned, this algorithm can be directly extended to any other specific spectrum masks because of its excellent flexibility. For example, if the target spectral mask is denoted by *M*(*f*)*,* then a UWB pulse can be generated from this design process only with *M_FCC_*(*f*) in (11) replaced by *M*(*f*). As a useful application, we take the Korean emission limit into the proposed algorithm [28]. Then, the designed UWB PSD has been shown in Figure 3(d). We note that although there are seven sharp stairs in this spectral constraint, our UWB pulse can still use the whole mask entirely. The obtained NESP of modified UWB pulse can even reach 92.67%. So our design algorithm can be widely applied to the most UWB spectrum masks with the *stairs* features, such as the regulatory UWB emission masks of Britain, Japan, Korea and Singapore [6,28,29]. It is also noted that a new UWB emission mask will be adopted after December 2010 [30], the corresponding UWB waveform is also shown in Figure 3(e).

**Figure 4.**
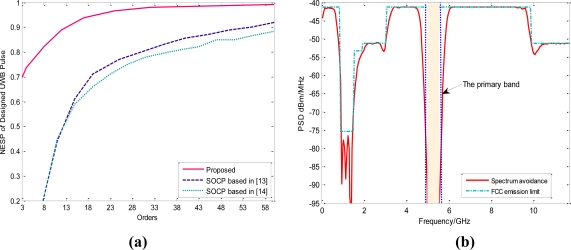
**(a)** Equivalent filter orders *vs.* NESP. **(b)** Designed UWB pulse with a spectrum notch in [5–5.5] GHz.

If the center and shape of each basis waveform **w̃***_i_* are optimized together with the weight vector **a** with the goal of minimizing the MSE according to the steepest descent algorithm [25], the NESP advantage of the proposed UWB waveform becomes rather obvious from Figure 4(a). To obtain the similar NESP, our desired filter orders are much smaller than those of the SOCP technique. Specifically, the required design orders of the proposed algorithm are only 15 when the NESP surpasses 90%, while the expected orders in [13] even reach 53. Therefore, the hardware implementation of our scheme is also much superior to SOCP based schemes considering the order of the baseband delay lines.

### 2.5. Spectrum Notch

As is indicated by recent investigations, the regulatory constraint on spectral limit is not safe enough for certain specific legal systems in many cases, such as the fixed wireless access (FWA) [31]. In a cognitive radio scene, the secondary UWB user should perform *spectrum avoidance* to eliminate its potential interference or the interference from other narrowband systems [15,20]. Hence, UWB waveforms should be also equipped with the flexibility of generating the spectrum notches to avoid the legal bands. Fortunately, our proposed scheme can effectively sculpt the spectrum under any given constraint [25,32].

Without loss of generality, assume that there is only one vulnerable service located in [5 5.5] GHz. With little effort, the corresponding sub weight vector, denoted by **a***_avoid_*, can be determined from (16) with *f_down_* and *f_up_* replaced by this vulnerable band. Then by directly resetting **a***_avoid_*, the UWB waveform with deep spectrum notch can be designed as is shown in Figure 4(b). It can be found that the attenuation in the legal band can even reach 50 dB. By comparison, the depth of such spectrum notches based on multi-band orthogonal frequency division multiplexing (MB-OFDM) is only about 20 dB, even if the specific coding techniques between the sub-carriers are adopted at the expense of undermining the spectrum efficiency [33]. The notch depth of the spectrum adaptive pulse based on Hermit-Gaussian function is only 25 dB [20]. Hence, our proposed UWB pulse is still much attractive if the *spectrum sculpting* technique is taken into consideration.

## 3. Design of Orthogonal UWB Pulse

From this two-phase design algorithm presented above, high NESP can be easily achieved under any emission constraint. Nevertheless, the designed waveforms are not mutually orthogonal, which has ruled out its significant applications in the multidimensional modulations and WDMA to further improve frequency efficiency. However, orthogonal pulses can be conveniently derived based on the proposed algorithm.

### 3.1. Orthogonality Constraint

In order to achieve waveform orthogonality and differentiate multiple users, a *characteristic code* should be assigned to the *i^th^* user at time *t*, which is denoted by **c***_i_*(*t*):

(23) $${\text{c}}_{i}\left(t\right)=\left[c_{i,0}\;c_{i,1}\;\cdots c_{i,N-1}\right]$$

where *c_i,p_* represents the *p^th^* element of the *i^th^* characteristic code. For convenience of analysis, here we assume the dimension of **W** is *N* × *N*. Then, the Fourier transform of the *i^th^* UWB orthogonal pulse at time *t* can be written as:

(24) $$S_{i}\left(k,t\right)={\text{c}}_{i}\left(t\right)⊗\left({\text{a}}_{\text{opt}}\text{W}\right)=\sum_{l=0}^{N-1}c_{i,l}\alpha_{l}{\tilde{\text{w}}}_{l}$$

Consequently, the time sequence *s_i_*(*n,t*) can be easily derived by IDFT on *S_i_*(*k,t*). If two users keep orthogonal, the correlation of their waveforms should satisfy:

(25) $$\sum_{n=0}^{N-1}s_{i}\left(n,t\right)s_{j}^{*}\left(n,t\right)=\{\begin{array}{ll} 1 & i=j\\ 0 & i\neq j \end{array}$$

By substituting the expression of *s_i_*(*n,t*) into Equation (25) and simplifying it, the cross correlation of the UWB pulses can be written as:

(26) $$\sum_{n=0}^{N-1}s_{i}\left(n\right)s_{j}^{H}\left(n\right)=\sum_{n=0}^{N-1}w\left(n\right)w^{H}\left(n\right)\left[\sum_{p=0}^{N-1}\sum_{q=0}^{N-1}\alpha_{p}c_{i,p}c_{j,q}^{H}\alpha_{q}^{H}\text{exp}\left(-j\frac{2\pi }{N}\text{ln}\left(p-q\right)\right)\right]$$

Combing the previous target of maximizing the NESP with the orthogonality constraint together, the general objective of the orthogonal waveform design is given by:

(27) $$\begin{array}{l} \text{min}\left\|{\left(\sum_{l=0}^{N-1}c_{il}\alpha_{l}w_{l}\right)}^{2}-M\left(k\right)\right\|\\ s.t.\;\sum_{p=0}^{N-1}\sum_{q=0}^{N-1}\alpha_{p}c_{i,p}c_{j,q}^{H}\alpha_{q}^{H}\text{exp}\left(-j\frac{2\pi }{N}\text{ln}\left(p-q\right)\right)=\{\begin{array}{ll} 1 & i=j\\ 0 & i\neq j \end{array} \end{array}$$

From (27), the design process can be viewed as an optimal problem subject to a specific constraint. An intuitive solution is to design the code set **c***_i_* based on the already optimized UWB pulses with maximum NESP, then we have:

(28) $$\{\begin{array}{l} \left|{\text{a}}_{i}\right|=\left\{\left|\alpha_{q}c_{i,q}\right|\right\}=\left|{\text{a}}_{\text{opt}}\right|,\;\left|{\text{a}}_{j}\right|=\left\{\left|\alpha_{q}c_{j,q}\right|\right\}=\left|{\text{a}}_{\text{opt}}\right|\\ \sum_{p=0}^{N-1}\sum_{q=0}^{N-1}\alpha_{p}c_{i,p}c_{j,q}^{H}\alpha_{q}^{H}\text{exp}\left(-j\frac{2\pi }{N}\text{ln}\left(p-q\right)\right)=\{\begin{array}{ll} 1 & i=j\\ 0 & i\neq j \end{array} \end{array}$$

where |*x*| denotes the amplitude value of *x*.

Equation (28) implies that the code set should fulfill *|c_i,q_|=1*. In the simplest case where there are only two UWB users transmitting signal simultaneously, we may choose the code set **c***_i_* (*i =* 0,1) directly as follows:

(29) $$\{\begin{array}{ll} c_{0,q}=1, & q=-N+1,\cdots ,-1,0,1,\cdots N-1\\ c_{1,q}=\text{sgn}\left(q\right)\times j, & q=-N+1,\cdots ,-1,0,1,\cdots N-1 \end{array}$$

where *sgn*(*x*) denotes the sign of *x*. It is apparent from Equation (29) that the designed *s*_0_(*n*) would be an even symmetry waveform while *s*_1_(*n*) is an odd symmetry one, and hence they keep mutually orthogonal.

With increasing prospective orthogonal users, the solution to (28) would become much more complicated. If the constraint is further weakened where the expectation of *overall NESP* during a long period is maximized, this problem becomes somewhat simple and the code set **c***_i_*(*T_k_*) is supposed to change with time *T_k_* to meet:

(30) $$\{\begin{array}{l} E\left[\left|{\text{a}}_{i}\right|\right]=\delta \left|{\text{a}}_{\text{opt}}\right|,\;\;\;\;\;\;E[\left|{\text{a}}_{j}|\right]=\delta \left|{\text{a}}_{\text{opt}}\right|\\ \sum_{p=0}^{N-1}\sum_{q=0}^{N-1}\alpha_{p}c_{ip}c_{jq}^{H}\;\alpha_{q}^{H}\;\text{exp}\left(-j\frac{2\pi }{N}\text{ln}\left(p-q\right)\right)=\{\begin{array}{ll} 1 & i=j\\ 0 & i\neq j \end{array} \end{array}$$

where *E*(**x**) gives the average value estimation of **x** from a long time; δ^2^ represents the bearable degradation on the already maximized NESP. If the code set **c***_i_*(*T_k_*) is further specified with its element chosen from {0,1}, the design process is equivalent to assigning frequency hopping (FH) patterns to a number of users at given time *T_k_*. Although orthogonal waveforms can be easily obtained, the momentary NESP of each user inevitably experiences an obvious decrement.

### 3.2. Orthogonality Design

From above analysis, the orthogonal waveforms design schemes directly in time domain are either complicated or suboptimal in NESP. Before reaching a more effective solution to Equation (28), nevertheless, we may consider another enlightening problem in frequency domain. Supposed each *S_i_*(*f*) represents the Fourier transform of a set of time waveforms *s_i_*(*t*):

(31) $$S_{i}\left(f\right)=A_{i}\left(f\right)\cdot \text{exp}\left(-j\theta_{i}\left(f\right)\right)$$

where *A_i_*(*f*) represents the amplitude response while θ*_i_*(*f*) is the phase response. It is well known that the correlation of two waveforms *R*_12_(0) is 0, when these two arbitrary pulses *s*_1_(*t*) and *s*_2_(*t*) keep mutually orthogonal. Then, if *the correlation property* of the Fourier transform is applied, we obtain:

(32) $$\frac{1}{2\pi }\int_{-\infty }^{\infty }S_{1}\left(f\right)S_{2}^{*}\left(f\right)df=0$$

Furthermore, if we let *A_i_*(*f*) *= K*, of particular importance is the phase response θ*_i_*(*f*) which can be carefully devised to attain mutually orthogonality among the waveform set *s_i_*(*t*). Substituting Equation (31) into (32), *R*_12_(0) can be simplify to *K*^2^ *×* ∫*_f_* exp[*-j*θ_1_(*f)*].exp[*j*θ_2_(*f*)]*df*. As a result, under the assumption that the amplitude response remains unchanged, the orthogonal waveforms design is converted to devising the appropriate phase responses θ_1_(*f*) and θ_2_(*f*) to meet:

(33) $$\int_{-\infty }^{\infty }\text{exp}\left(-j\theta_{1}\left(f\right)\right)\cdot \text{exp}\left(j\theta_{2}\left(f\right)\right)df=0$$

To reduce the complexity of (33), we may further specify the phase response θ*_i_*(*f*) in the following form:

(34) $$\theta_{i}\left(f\right)=\frac{1}{4}c_{i}\left(f\right),\;\;\;c_{i}\left(f\right)\in \left\{+1,-1\right\}$$

Then, Equation (32) will be translated into ∫*_f_c*_1_(*f*)*c*_2_(*f*)*df=*0. In this situation, orthogonality can be easily satisfied if we substitute the orthogonal pseudorandom code for **c***_i_*, such as the maximum length binary sequence. For the sample based Fourier transform sequence *S*(*k*) (*k =* 0,1,…, *N-*1), the above conclusion is also straightforward. The expected maximum size of orthogonal waveform set 2^⌊log_2_^ *^N^*^⌋^ is when the length of *S*(*k*) is *N*.

By now, we can come back to the general solution to the spectrally efficient orthogonal waveform design in Equation (28). Considering that the regulated UWB emission limit can be always viewed as a piecewise flat function [6,8–30], we may divide the whole mask into multiple pieces of spectral lines with *constant amplitude* so the above conclusion can be conveniently applied. Directly, the FCC spectral constraint can be expressed as a combination of six sectional PSD lines *M*^(^*^k^*^)^(*f*)*, k =* 1,2,3,4,5,6:

(35) $$M_{\text{FCC}}^{\prime }\left(f\right)=\sum_{k=1}^{6}M^{\left(k\right)}\left(f\right)$$

where *M*^(^*^k^*^)^(*f*) represents one piece of spectral line locating at non-overlapping frequency band. In order to fully utilize the authorized band, the orthogonal UWB pulse should occupy the total six sections of spectral line *M*^(^*^k^*^)^(*f*) to which an orthogonal sequence **c***_i_*^(^*^k^*^)^(*f*) is assigned. Accordingly, the allowable orthogonal UWB users *Num_th* should be determined by the narrowest band:

(36) $$\text{Num}\_\text{oth}=\text{min}\left\{2^{\left\lfloor {\text{log}}_{2}\left(N^{\left(i\right)}\right)\right\rfloor },i=1,2,3,4,5,6\right\}$$

where ⌊*x*⌋ denotes the round integer of *x; N*^(^*^k^*^)^ represents the length of the *k^th^* piece spectral line *M*^(^*^k^*^)^(*f*). If we represent *S*(*k*) by *N* samples, then the maximum orthogonal users are about 2^⌊log_2_^ *^N^*^⌋^ – 2^⌈log_2_ (12.5/0.38)⌉^, which exactly corresponds to the narrow band occupying 1.61–1.99 GHz. The maximum orthogonal waveform set is only eight when *N* is 256.

To enlarge this orthogonal set, it is necessary to modify FCC mask with the slightest discrepancy from *M_FCC_*(*f*). This process is mainly to eliminate the extremely narrow band without causing a much significant degradation on NESP. A feasible modified FCC spectral mask can be written as:

(37) $$M_{\text{FCC}}^{\prime }\left(f\right)=\{\begin{array}{ll} -75.3dB & f\in \left[0,\;\;1.99\right]GHz\\ -53.3dB & f\in \left[1.61,\;\;3.1\right]GHz\\ -41.3dB & f\in \left[3.1,\;\;10.6\right]GHz\\ -51.3dB & f\in \left[10.6,\;\;+\infty \right]GHz \end{array}$$

**Figure 5.**
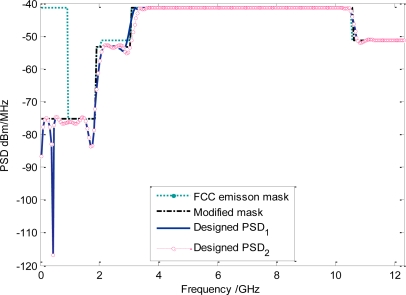
Modified UWB emission limit. Note that PSD_1_ represents designed PSD from (37), whereas PSD_2_ extends its second piece spectral line *M*^(2)^(*f*) based on PSD_1_.

The designed *M*’*_FCC_*(*f*) which is denoted by PSD_1_ has been shown in Figure 5. In such a case, the corresponding maximum orthogonal UWB users are 16, according to Equation (36). If we further extend *M* ^(2)^(*f*) a little bit to 3.2 GHz, then the maximum orthogonal set can be improved to 32. The corresponding designed PSD_2_ has also been illustrated in Figure 5. Additionally, the maximum orthogonal users can be enlarged with the increasing of the samples length of *M_FCC_*(*f*); for example, the total orthogonal UWB users can even reach 64 when the total samples of *M_FCC_*(*f*) are 512.

In Equation (37), we have decomposed the UWB emission mask based on the frequency axis. Alternatively, we may also break it mask into multiple pieces of constant spectral lines from the magnitude view. Another decomposition of the FCC spectral mask can be expressed as:

(38) $$M_{\text{FCC}}^{\prime \prime }\left(f\right)=\sum_{i=1}^{3}M^{\left(i\right)}\left(f\right)$$

where *M*^(^*^k^*^)^(*f*) is a piece of spectral line which overlaps each other in certain frequency range:

(39) $$\{\begin{array}{ll} M^{\left(1\right)}\left(f\right)=-75.3dB & f\in \left[1.99,\;\;10.6\right]GHz\\ M^{\left(2\right)}\left(f\right)=-54.02dB & f\in \left[1.61,\;\;10.6\right]GHz\\ M^{\left(3\right)}\left(f\right)=-43.8dB & f\in \left[3.1,\;\;10.6\right]GHz \end{array}$$

Correspondingly, the *orthogonal variable spreading factor* (OVSF) sequence can be used as the orthogonal code in Equation (34). In such a case, the maximum orthogonal set can be extended to 64 when *N* is chosen to 128, which is determined by *M*^(3)^(*f*).

### 3.3. Orthogonality Analysis

With the aid of the proposed shaping filter in Figure 2, the orthogonality design is straightforward. Depending on different spectral mask partition patterns in Equations (37) and (39), the optimal spectrum *S_even_*(*k*) is multiplied by two kinds orthogonal sequence respectively. Given that the PSD is only related with the amplitude response *A*(*f*), there would be few degradations on the NESP of our designed orthogonal UWB pulses.

Practically, the cross correlation is not zero because the designed amplitude response can hardly remain constant during each spectral line. If we denote the error between the ideal spectrum and the actual output spectrum by *o*(*f*) and constant amplitude by *K*, then the correlation of the orthogonal pulses can be written as:

(40) $$\begin{array}{l} R_{c}=\int_{f_{B}}\left[K-o\left(f\right)\right]C_{1}\left(f\right)\times \left[K-o\left(f\right)\right]C_{2}\left(f\right)df\\ \;\;\;\;\;\;=\left|K^{2}\int_{f_{B}}C_{1}\left(f\right)C_{2}\left(f\right)df-2K\int_{f_{B}}o\left(f\right)C_{1}\left(f\right)C_{2}\left(f\right)df+\int_{f_{B}}o^{2}\left(f\right)C_{1}\left(f\right)C_{2}\left(f\right)df\right| \end{array}$$

Considering that the high-order error part *o*^2^(*f*) is extremely small, we can discard the third term from the right side of Equation (40). The first term represents the correlation of the *m* sequence. Usually, the second term is larger than the first one. If we further normalize the correlation by its autocorrelation, Equation (40) can be expressed into:

(41) $$\begin{array}{ll} R_{c} & =2K\frac{\left|\int_{f_{B}}o\left(f\right)C_{1}\left(f\right)C_{2}\left(f\right)df\right|}{\int_{f_{B}}{\left|KC_{1}\left(f\right)\right|}^{2}df}-\kappa \\ & \leq 2\frac{\sqrt{\int_{f_{B}}{\left|o\left(f\right)C_{1}\left(f\right)\right|}^{2}df\times \int_{f_{B}}{\left|KC_{2}\left(f\right)\right|}^{2}df}}{\int_{f_{B}}{\left|KC_{1}\left(f\right)\right|}^{2}df}-\kappa \approx 2\sqrt{\left(1-\text{NESP}\right)}-\kappa \end{array}$$

where κ is the normalized correlation of *m* sequence. Basically, according to Hölder inequality [34], the derived result in (41) can never be achieved so this analysis only provides the *upper bound* of correlation values. From (41), the maximum correlation is close related to the designed NESP, and a higher NESP means the smaller cross correlation.

**Figure 6.**
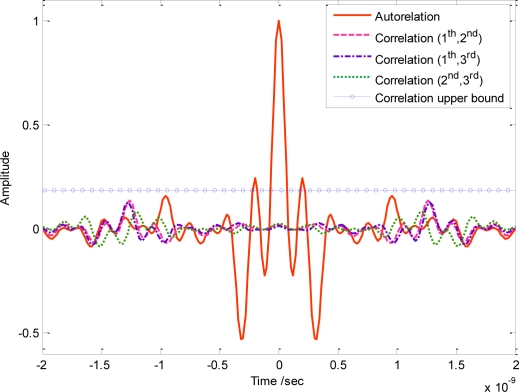
Correlation of orthogonal UWB waveforms based on (37). The corresponding autocorrelation is normalized, whereas the cross correlation is about 1.97 × 10^−3^.

Without loss of generality, we assume that there are three users transmitting signals simultaneously in UWB sensor networks. The autocorrelation and correlation of these three orthogonal waveforms have been illustrated in Figure 6. The autocorrelation has been normalized; however, the maximum correlation is about 1.97 × 10^−3^, even with perfect synchronization. The mainly reason lies in that the actual designed PSD in Figure 5 is quite smooth near the sharp discontinuous edges, which has slightly violated the ideal assumption that the spectral line remains a constant. According to analysis, the upper bound of the correlation is about 0.2 when NESP reaches 98.7%, as is shown by the dot line in Figure 6. Actually, in our simulation, the maximum κ of adopted orthogonal sequences is about 0.04. As a result, according to (41), the correlation upper bound based on the numerical simulation is about 0.2.

**Figure 7.**
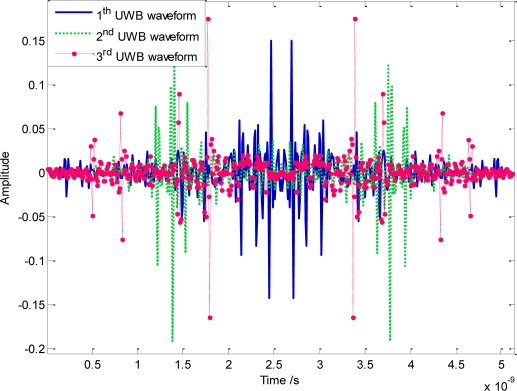
Time domain waveforms for orthogonal UWB pulses.

It is also noteworthy that, attributed to the combination concept of several narrow-band signals that have no mutual phase constraints, the orthogonal signal generated from Equation (35) or (38) may be considerably broadened in the time domain. The corresponding orthogonal waveforms have also been plotted in Figure 7. For a WDMA based UWB sensor network, therefore, transmission data rate will be restricted to some extent, in order to avoid the inter symbol interference (ISI) caused by orthogonal waveforms with a long duration. Notice that, since the orthogonality designing scheme mainly alters the phase response of UWB signals, the frequency domain amplitude shapes for all waveforms may basically remain the same as that seen in Figure 5.

## 4. Performance Evaluation

In this part, we evaluate the performance of our proposed scheme in a waveform division multiple access (WDMA) network.

### 4.1. Performance with Accurate Timing

The achieved SNR is proportional to the emission power when the template-matched demodulation has been employed in receiver [34]. For the purpose of analysis, we assume that the path loss here is 0 dB and the noise is additive Gaussian white noise (AWGN). Hence, the output SNR with accurate timing can be written as:

(42) $$P_{e}=\frac{1}{2}\text{erfc}\left(\sqrt{\bar{\text{NESP}}\times \frac{\int_{f_{B}}M_{\text{FCC}}\left(f\right)df}{\int_{f_{B}}P_{n}\left(f\right)df}}\right)=\frac{1}{2}\text{erfc}\left(\sqrt{\bar{\text{NESP}}\times \frac{R_{a}\left(0\right)}{\int_{f_{B}}P_{n}\left(f\right)df}}\right)$$

where *P_n_*(*f*) represents the PSD of channel noise, and *R_a_*(0) is the autocorrelation of the ideal UWB pulse. For the *ideal* UWB pulses with NESP = 1, the obtained SNR under the given *P_n_*(*f*) is defined as the *target SNR*. Then the difference between the actually output SNR in UWB receiver and the target SNR can be defined as the *margin SNR,* which can used to distinctly evaluate BER performance of different UWB waveforms.

**Figure 8.**
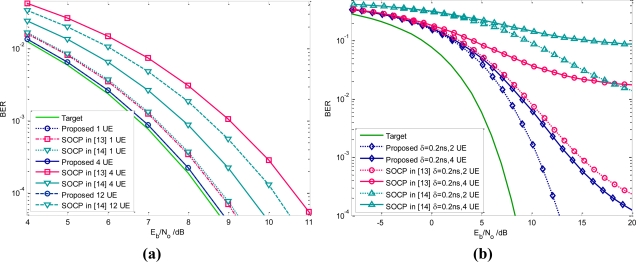
**(a)** Performance with accurate timing. **(b)** Performance with the synchronization error. Note that the filter orders of both the proposed method and SOCP scheme are 32.

In our following simulation, the parameters are set as the same in Section 2. Table 1 shows the NESP of the existing UWB waveforms. The obtained NESP for single orthogonal pulses is about 98.7% under FCC spectral constraint. So this designed pulse slightly outperforms the SOCP based UWB pulse which is about 92.16% [13]. During the BER evaluation, the uncoded binary PAM is adopted in transmitter and the coherent correlator is employed to perform optimal receiving. A careful observation of Figure 8(a) indicates that, for single user, the *margin SNR* of the proposed pulse is 0.1 dB and the SOCP based UWB pulse is 0.4 dB.

When it comes to multiple orthogonal UWB pulses, we should employ the average NESP to judge the transmission performance. From Table 1, the designed average NESP of the Hermite-Gaussian based orthogonal pulse is only 65% [20], and the wavelet based one is about 78.4% [21]. Although the SOCP scheme is suitable for a single UWB waveform, the NESP of subsequent generated orthogonal pulse of the sequential algorithm in [13] experiences an obvious degradation.

For example, the NESP of the first obtained pulse is 76.51%, and that of the second designed pulse is only 51.31%. As a result, the average NESP is only 59.26% for three UWB users. Based on this sequential solving scheme, the design algorithm is also much more complicated than our algorithm. The average NESP of another SOCP based orthogonal pulse design algorithm in [14] is about 77.5% (four orthogonal users). By comparison, our proposed algorithm can generate mutual orthogonal pulses without any penalty on NESP. The average NESP can reaches 96.7% under FCC mask and 98.7% under the modified mask. So, it is clear that our orthogonal pulses outperform the other schemes in WDMA. If accurate timing is acquired in UWB receiver, from simulations illustrated in Figure 8(a), the BER performance of the proposed waveforms, in four users WDMA network, can surpass the SOCP method in [13] about 2 dB. We may reasonably deduce that the superiority of the proposed pulse in WDAM becomes much more apparent with increasing of the number of orthogonal users.

### 4.2. Performance with Synchronization Deviation

As far as the waveform division multiple access networks are concerned, in practice, the synchronization deviation caused by the devices movement or clock drift may dramatically worsen receiving performance. Supposed the maximum timing deviation is δ, the BER performance with *M* users is given by:

(43) $$P_{e}=\frac{1}{2}\text{erfc}\left(\sqrt{\bar{\text{NESP}}\times \frac{R\left(\delta \right)}{MR_{c}\left(\delta \right)+\int_{f_{B}}P_{n}\left(f\right)df}}\right)$$

where *R_c_(*δ*)* is the cross correlation of orthogonal UWB users. Based on numerical computations, the BER performance for two users and for four users have been shown in Figure 8(b). Evidently, our proposed orthogonal pulses have a great advantage over the SOCP pulses when there is timing inaccuracy in UWB receiver. Specifically, our pulses can obtain about 9 dB gain compared to the SOCP based orthogonal pulses in [13] when the maximum deviation is 0.2 ns and the orthogonal users in WDMA sensor networks are two. At the same time, the SOCP pulses in [14] have the worst BER performance because of their correlation characteristics. Therefore, from aspect of the practical applications, our scheme can reduce the stringent requirement on synchronization, and hence simplify the receiver complexity [27].

### 4.3. Other Considerations

It should also be emphasized that, in addition to the distinguished transmission performance in WDMA, this proposed algorithm has some other mentionable merits. The implementation of our UWB pulse is much more competitive. The baseband processing frequency is only about 7 GHz when the total occupying band is 12.5 GHz, which is substantially smaller than the desired baseband sampling rate of 28 GHz in the SOCP method. The design orders are also far less than that of the SOCP scheme given an expected NESP. Based on cyclic shift and FFT, our proposed structure also has the virtues of simple implementations compared to pulse generator which employs dozens of carrier synthesizers.

Besides, this goal-directed algorithm provides great reconfigurability to any specific pulse design, which makes our scheme a general signal generator given an arbitrary spectral shape. As a useful application, we have designed UWB pulse with satisfactory frequency utilization under some regulatory spectral constraints. This method also paves the way for the *underlay* application of UWB in cognitive radio. The cognitive waveforms with arbitrary spectrum notch can be easily generated to substantially eliminate the potential interference to primary users. The attenuation of the corresponding spectrum notch can even reach 50 dB, which is much superior to other proposals such as the OFDM based cognitive transmission strategy. Finally, it is vital to mention that this kind of spectrum notch can be effectively used for interference mitigation from other narrow band systems, thereby improving transmission performance of UWB devices.

### 4.4. Realistic Front-End Effects

**Figure 9.**
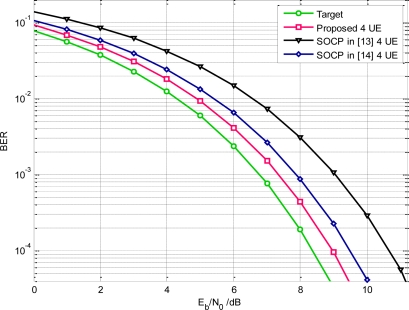
BER performance by taking consideration of realistic UWB antennas.

Throughout the above discussions the ideal UWB antenna is assumed, which exhibits a flat amplitude frequency (AF) response in a large range which covers from the DC frequency to 12.5 GHz. Nevertheless, in practice, the realistic front-end amplifier or UWB antenna acts as the band-pass filters that can only utilize a part of authorized spectrum, generally focused on the FCC regulated 3.1–10.6 GHz band. Thus, the well-designed UWB waveform will be further filtered by these non-ideal devices. As a result, the effort put into waveform design that occupies the FCC approved spectrum below 960 MHz may have little actual impact on the increase of receiver SNR. Specifically, the achieved NESP in the whole FCC band will be decreased to 87.6%, for the single user, which is slightly superior to the SOCP based UWB waveform with a NESP of 82.08% in [13]. Correspondingly, the achieved SNR gain will also be reduced to some extent. The average BER performance of a WDMA network with four UWB users is illustrated in Figure 9 by taking the realistic nonideal front-end devices into account, from which we may observe that the receiving performance gain indeed decrease compared to Figure 8(a). Nevertheless, less basis functions are required in this situation leading to a much simpler implementation.

If the AF response of realistic UWB antennas remains flat in this partial frequencies range, then the number of the orthogonal UWB users may not be determined by the narrow spectral lines any more. Similarly, if we represent *S*(*k*) with *N* samples, then the maximum orthogonal users changes to 2^⌊log_2_ N⌋^, since the used basis waveforms in this case are mainly located in occupied band of 3.1–10.6 GHz, so it can provide much more UWB users to simultaneously access to the spectrum, accompanying the simplified processing. On the other hand, if the non-flat AF response of the realistic front-end is taken into consideration, the validity of the presented orthogonality derivation for multiple UWB waveforms under the ideal RF device assumption may be lost. As a result, except for the performance degradation in UWB receivers, even the mutual orthogonality of multiple users could be destroyed.

As a simply potential solution to still keep the orthogonality of designed waveforms and also enhance receiving SNR even in the presence of non-plat UWB antennas, the *waveform predistortion* technique can be introduced into our original design algorithm [36]. Specifically, given the frequency response of the generalized front-end devices denoted by *G*(*f*), then the designing EIRP target in (11) can be slightly modified into *M*(*f*)*/G*(*f*). It is obvious that, after the designed pulse passed the realistic antenna *G*(*f*), then the filtered UWB waveforms still keep *flat* in their authorized working bands. Therefore, the mutual orthogonality can be still guaranteed in this case. Actually, it is apparent that the original designing algorithm can be regarded as a special case of this general scheme, in which we ideally assume *G*(*f*) = 1. This detailed predistortion algorithm will be further investigated comprehensively in our near future research.

## 5. Conclusions

Although UWB-IR techniques show many attractive features in short-range high-data-rates communication as well as in other important applications, electromagnetic compatibility (EMC) between UWB sensors and the other vulnerable wireless systems sharing the same band should be carefully investigated. It is encouraging to see that many countries have already regulated UWB emission limits according to their own practical situations, which lays the foundation for widespread applications of UWB. Since the UWB emission limit always remains below −41.3 dBm/MHz, the UWB transmitted pulses should fully make use of authorized spectral energy to enhance the SNR in receiver. At the same time, in order to provide the orthogonal waveform diversity and mitigating the mutual interference between UWB sensors, orthogonality is also worthy to be included in the waveforms designing.

In this paper, we have presented a versatile UWB waveform from the transform domain. Although FCC spectral mask is taken as an example to design the UWB signal, our algorithm can actually be used for any spectral mask. Compared with the other existing UWB spectrum forming techniques, such as the FIR shaper and the pulse optimization, our proposed optimal algorithm is considerably simpler in realization and superior in NESP. Based on this suggested algorithm, the obtained UWB waveform with specific spectrum notches also has an important application in CR networks. What’s more, orthogonal pulses can be easily derived from the presented scheme without any degradation on NESP, which can be suitably applied in UWB systems to improve the frequency efficiency. The generated mutually orthogonal waveforms are also much more competitive than other schemes in a multiuser scene, especially when the WDMA sensor networks can not acquire accurate synchronization.

## Figures and Tables

**Table 1.** NESP of the existing UWB signals under FCC mask Note that the filter orders of both the proposed method and SOCP scheme are 32. The average NESP is evaluated under the modified FCC mask. Assume that the total orthogonal users are 4; however, the average NESP in [13] is based on 3 users.

**Different techniques**	**NESP for single pulse**	**Ave. NESP for orthogonal pulses**
Seventh derivative Gaussian pulse [35]	46%	-
Hermite Gaussian Functions [20]	65%	65%
PM algorithm [10]	72.41%	-
SOCP based FIR filter [14]	79%	77.5%
SOCP based FIR filter [13]	92.16%	59.26%
Proposed UWB pulse	98.7%	98.7%
